# A Mn-N_3_ single-atom catalyst embedded in graphitic carbon nitride for efficient CO_2_ electroreduction

**DOI:** 10.1038/s41467-020-18143-y

**Published:** 2020-08-28

**Authors:** Jiaqi Feng, Hongshuai Gao, Lirong Zheng, Zhipeng Chen, Shaojuan Zeng, Chongyang Jiang, Haifeng Dong, Licheng Liu, Suojiang Zhang, Xiangping Zhang

**Affiliations:** 1 Beijing Key Laboratory of Ionic Liquids Clean Process, State Key Laboratory of Multiphase Complex Systems, Institute of Process Engineering, Chinese Academy of Sciences, 100190 Beijing, P.R. China; 2 College of Chemical Engineering, University of Chinese Academy of Science, 100049 Beijing, P.R. China; 3 Beijing Synchrotron Radiation Facility (BSRF), Institute of High Energy Physics, Chinese Academy of Sciences, 100049 Beijing, P.R. China; 4 Dalian National Laboratory for Clean Energy, 116023 Dalian, P.R. China; 5 Qingdao Institute of Bioenergy and Bioprocess Technology, Chinese Academy of Sciences, 266101 Qingdao, P.R. China

**Keywords:** Electrocatalysis, Electrocatalysis, Electrocatalysis

## Abstract

Developing effective catalysts based on earth abundant elements is critical for CO_2_ electroreduction. However, simultaneously achieving a high Faradaic efficiency (FE) and high current density of CO (*j*_CO_) remains a challenge. Herein, we prepare a Mn single-atom catalyst (SAC) with a Mn-N_3_ site embedded in graphitic carbon nitride. The prepared catalyst exhibits a 98.8% CO FE with a *j*_CO_ of 14.0 mA cm^−2^ at a low overpotential of 0.44 V in aqueous electrolyte, outperforming all reported Mn SACs. Moreover, a higher *j*_CO_ of 29.7 mA cm^−2^ is obtained in an ionic liquid electrolyte at 0.62 V overpotential. In situ X-ray absorption spectra and density functional theory calculations demonstrate that the remarkable performance of the catalyst is attributed to the Mn-N_3_ site, which facilitates the formation of the key intermediate COOH^*^ through a lowered free energy barrier.

## Abstract (web summary)

Developing effective catalysts based on earth abundant elements is critical for electrochemical CO_2_ reduction. Here, the authors prepare a manganese single atom catalyst with high CO_2_ reduction performance, which can be attributed to the Mn-N_3_ site embedded in graphitic carbon nitride.

## Introduction

Electrochemical CO_2_ reduction reaction (CO_2_RR) is one of the most promising ways to relieve CO_2_ accumulation^1,2^. Nevertheless, the CO_2_RR process involves multiple proton–electron transfer reactions, and the H_2_ evolution reaction (HER) always competes with it. Novel catalysts are strongly desirable to simultaneously obtain a high Faradaic efficiency (FE) and high current density of target products^3^. Among the various products of CO_2_RR, CO is one of the most practical targets, since it can be used to prepare synthetic fuels and chemicals via a downstream Fischer–Tropsch process^4–7^. Electrocatalysts based on manganese (Mn), the third most abundant transition metal in Earth’s crust, have been reported as catalysts for the CO_2_RR, including Mn oxides^8^, Mn complexes^9^, Mn single-atom catalysts (SACs)^10–13^, and so on. Among them, Mn SACs have attracted great interest. However, the reported Mn SACs often use graphene as a substrate to form the Mn–N_4_ structure, and the performance for CO_2_RR is greatly limited, especially with regard to the CO partial current density (*j*_CO_). Strasser et al. fabricated a family of SACs (M–N–C) with a variety of transition metals, including Mn–N–C which exhibited only 65% CO FE with a *j*_CO_ of 3.3 mA cm^−2^ ^10^. Zhang et al. reported a new Mn SAC with halogen and nitrogen dual coordination ((Cl, N)–Mn/G) and found that it is an efficient strategy to improve CO_2_RR performance through controlling coordination of the active site by additional halogen in the SAC. The (Cl, N)–Mn/G showed a high CO FE of 97% at an overpotential of 0.49 V, while the *j*_CO_ was 9.2 mA cm^−2^ ^11^. Therefore, it is significant to synthesize new Mn SACs with high *j*_CO_ and CO FE.

Herein, we prepare a Mn SAC with a Mn–N_3_ site embedded in graphitic carbon nitride (g-C_3_N_4_) on carbon nanotubes (CNTs) for efficient CO_2_RR, which is denoted as Mn–C_3_N_4_/CNT. The prepared catalyst displayed a 98.8% CO FE with a *j*_CO_ of 14.0 mA cm^−2^ at a low overpotential of 0.44 V in aqueous electrolyte, which outperforms all Mn SACs reported in the literature. Interestingly, the *j*_CO_ was further improved in an ionic liquid (IL) electrolyte. In situ X-ray absorption spectra and density functional theory (DFT) calculations were conducted to investigate the process of CO_2_ adsorption, activation and conversion over Mn–C_3_N_4_/CNT, demonstrating that three N atoms coordinated to a Mn center in g-C_3_N_4_ can greatly improve the performance of the Mn SAC in the CO_2_RR. This work shows a means to enhance the CO_2_RR performance of Mn-based catalysts under mild conditions.

## Results

### Synthesis and structural characterizations of Mn–C_3_N_4_/CNT

**Fig. 1:**
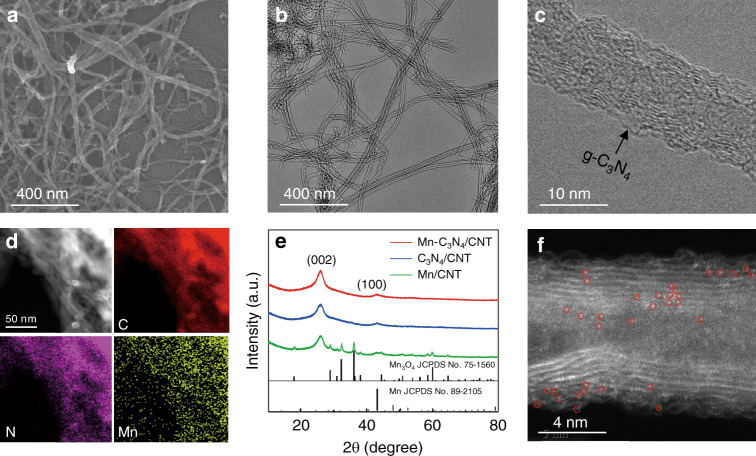
Structural characterizations of Mn–C_3_N_4_/CNT. **a** SEM image of Mn–C_3_N_4_/CNT. **b** Large-field view and **c** magnified view of TEM images of Mn–C_3_N_4_/CNT. **d** EDS mapping of Mn–C_3_N_4_/CNT. **e** XRD patterns of Mn–C_3_N_4_/CNT, C_3_N_4_/CNT, and Mn/CNT. **f** HAADF-STEM image of Mn–C_3_N_4_/CNT with the atomic dispersion of Mn highlighted by red circles.

Mn–C_3_N_4_/CNT was synthesized through thermal pyrolysis of Mn acetate, CNTs, and dicyandiamide (DCD) under nitrogen at 873 K followed by hydrochloric acid washing, and the addition of CNTs served to improve the g-C_3_N_4_ conductivity. Scanning electron microscopy (SEM) and transmission electron microscopy (TEM) images show that Mn–C_3_N_4_/CNT maintains the morphology of the CNTs without the formation of obvious particles (Fig. 1a, b). A thin enveloping layer of g-C_3_N_4_ (confirmed by powder X-ray diffraction (XRD) and X-ray photoelectron spectroscopy (XPS)) is observed on the surface of CNTs at a higher magnification (Fig. 1c). Energy-dispersive X-ray spectroscopy (EDS) images reveal that Mn, N, and C are homogeneously distributed on the entire surface of Mn–C_3_N_4_/CNT (Fig. 1d). For comparison, C_3_N_4_/CNT and Mn/CNT were synthesized by the same procedure as Mn–C_3_N_4_/CNT except for the omission of Mn acetate and DCD, respectively. It is worth pointing out that Mn/CNT was not treated by acid washing, as Mn was removed after acid washing, leading to inactivation of the catalyst. The morphologies of C_3_N_4_/CNT and Mn/CNT are almost identical except that there are obvious particles on Mn/CNT (Supplementary Figs. 1–3). The main peaks in the XRD pattern of Mn/CNT can be indexed as the Mn_3_O_4_ phase (JCPDS Card No. 75-1560), indicating the formation of a Mn_3_O_4_/CNT composite (Fig. 1e). For Mn–C_3_N_4_/CNT and C_3_N_4_/CNT, peaks at 25.8° and 42.8° can be ascribed to the (002) and (100) facets of the CNTs^14^, respectively, and no peak of g-C_3_N_4_ is observed. However, a peak appears at 27.4° after the amount of DCD is increased (Supplementary Fig. 4), which can be indexed to the interplanar stacking of aromatic systems of g-C_3_N_4_^15^. Therefore, the absence of g-C_3_N_4_ peaks in the Mn–C_3_N_4_/CNT XRD pattern is because they are covered by the high-intensity CNT peaks. Meanwhile, the absence of metallic peaks indicates that there are no crystalline metal-containing phases or clusters, which is in good agreement with the TEM results. The aberration-corrected HAADF-STEM image confirms the existence of atomically dispersed Mn in Mn–C_3_N_4_/CNT (Fig. 1f), and the Mn content is 0.17 wt% as measured by inductively coupled plasma atomic emission spectroscopy. The contents of N and C detected by elemental analysis are 9.8 and 81.1 wt%, respectively. Moreover, the specific surface areas of C_3_N_4_/CNT (271 cm^2^ g^−1^) and Mn–C_3_N_4_/CNT (221 cm^2^ g^−1^) are larger than that of Mn_3_O_4_/CNT (188 cm^2^ g^−1^), indicating that the thin enveloping layer of g-C_3_N_4_ on the surface of CNTs can increase the specific surface area (Supplementary Fig. 5).

### Fine structure of Mn–C_3_N_4_/CNT

**Fig. 2:**
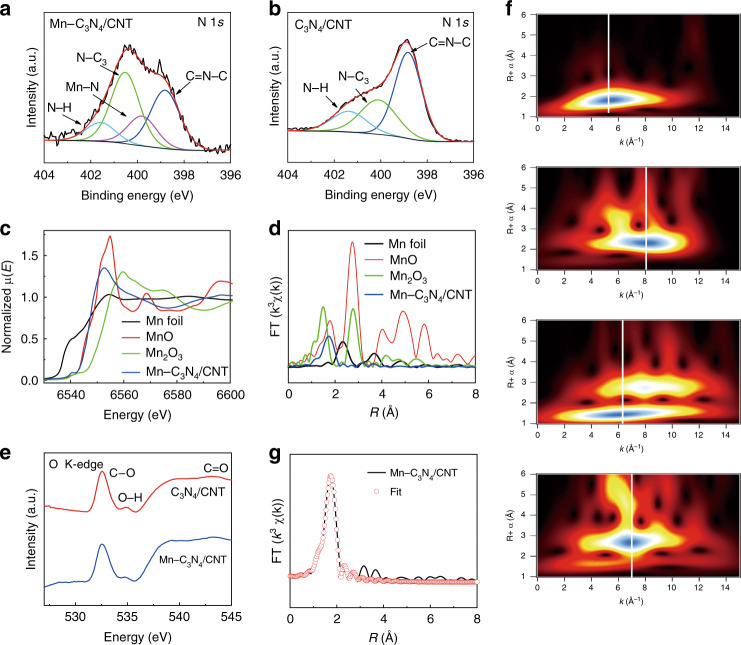
Fine structure of Mn–C_3_N_4_/CNT. **a**, **b** High-resolution N 1*s* XPS spectra of Mn–C_3_N_4_/CNT and C_3_N_4_/CNT, respectively. **c**, **d** XANES and EXAFS spectra at the Mn K-edge. **e** O K-edge XAS of Mn–C_3_N_4_/CNT and C_3_N_4_/CNT. **f** WT of Mn–C_3_N_4_/CNT, Mn foil, Mn_2_O_3_, and MnO (from top to bottom). **g** EXAFS fitting curves of Mn–C_3_N_4_/CNT in R space.

The chemical composition and elemental state of Mn–C_3_N_4_/CNT, C_3_N_4_/CNT, and Mn_3_O_4_/CNT were examined by XPS. The full XPS survey of Mn–C_3_N_4_/CNT scan shows the presence of Mn, N, C, and O (Supplementary Fig. 6). The existence of O is attributed to C = O or C–O groups on the surface of CNTs. The high-resolution XPS N 1*s* spectrum of Mn–C_3_N_4_/CNT (Fig. 2a) can be divided into triazine rings (C–N–C, ~398.9 eV, 33.6%), Mn–N (~399.8 eV, 17.0%), tertiary nitrogen (N–(C)_3_, ~400.4 eV, 38.6%), and amino functions (N–H, ~401.6 eV, 10.8%) of g-C_3_N_4_^16^. Compared with that of the N 1*s* spectrum of C_3_N_4_/CNT (Fig. 2b), the percentage of Mn–N increases from 0% to 17.0%, and the percentage of C–N–C decreases by ~15.4%, suggesting that C–N–C is most likely to provide coordination sites for Mn atoms to form Mn–N_*x*_ moieties. The XPS Mn 2*p* spectra of Mn–C_3_N_4_/CNT and Mn_3_O_4_/CNT are displayed in Supplementary Fig. 7, and the Mn 2*p*_3/2_ spectrum of Mn_3_O_4_/CNT is divided into two kinds of valence states, located at 641.2 and 642.9 eV, ascribed to Mn^2+^ and Mn^3+^, respectively^17^. The Mn 2*p*_3/2_ peak of Mn–C_3_N_4_/CNT is close to Mn^2+^, suggesting that the valence state of the Mn atom in Mn–C_3_N_4_/CNT is likely to be +2. The local structure of the catalyst at the atomic level was further determined by X-ray absorption spectroscopy (XAS). For the Mn K-edge X-ray absorption near-edge structure (XANES, Fig. 2c), the absorption edge of Mn–C_3_N_4_/CNT is close to that of MnO, located between Mn foil and Mn_3_O_4_, which further suggests that the valence state of Mn in Mn–C_3_N_4_/CNT is close to +2. The formation of Mn–N bonds in Mn–C_3_N_4_/CNT is directly confirmed by the phase-uncorrected Fourier transformed (FT) extended X-ray absorption fine structure (EXAFS) characterization (Fig. 2d). Mn foil presents the main peak at 2.3 Å, corresponding to the scattering path of Mn–Mn^16,18^. For Mn–C_3_N_4_/CNT, no Mn–Mn bond peak is detected, indicating atomically dispersed Mn. For the EXAFS spectrum of Mn–C_3_N_4_/CNT, the peak at 1.7 Å can be ascribed to Mn–N or Mn–O. For the XAS spectrum of the O K-edge, the Mn–O peak is not detected, and no difference in position between Mn–C_3_N_4_/CNT and C_3_N_4_/CNT (Fig. 2e) is found. Further considering the XPS results that the N atom of C–N–C provides a coordination site to form the Mn–N structure, the peak at 1.7 Å is ascribed to the scattering path of Mn–N^18^. The wavelet transform (WT) results further verify that Mn–Mn bonds are not present in Mn–C_3_N_4_/CNT, and the peak of Mn–C_3_N_4_/CNT tends to have a lower *k* value than that of Mn–O in Mn_2_O_3_ and MnO, indicating that the Mn–N bond is more likely to exist in Mn–C_3_N_4_/CNT (Fig. 2f). Quantitative EXAFS curve fitting analysis (Fig. 2g and Supplementary Table 1) was performed to investigate the structural parameters of Mn–C_3_N_4_/CNT, and the best-fitting analysis clearly confirms that the Mn–N coordination number is ~3.2, meaning that the isolated Mn atom is threefold coordinated by N atoms. The calculated Mn–N mean bond distance in Mn–C_3_N_4_/CNT is 2.21 Å which is longer than that of the Mn–N_4_ (2.02 Å) structure^11^. The results of the best-fitting analysis demonstrate that the coordination environment of the Mn atom in Mn–C_3_N_4_/CNT is different from that of Mn SACs reported in the literature^10,12,13^, which may cause different CO_2_RR performance.

### Electrochemical activities of CO_2_ reduction

**Fig. 3:**
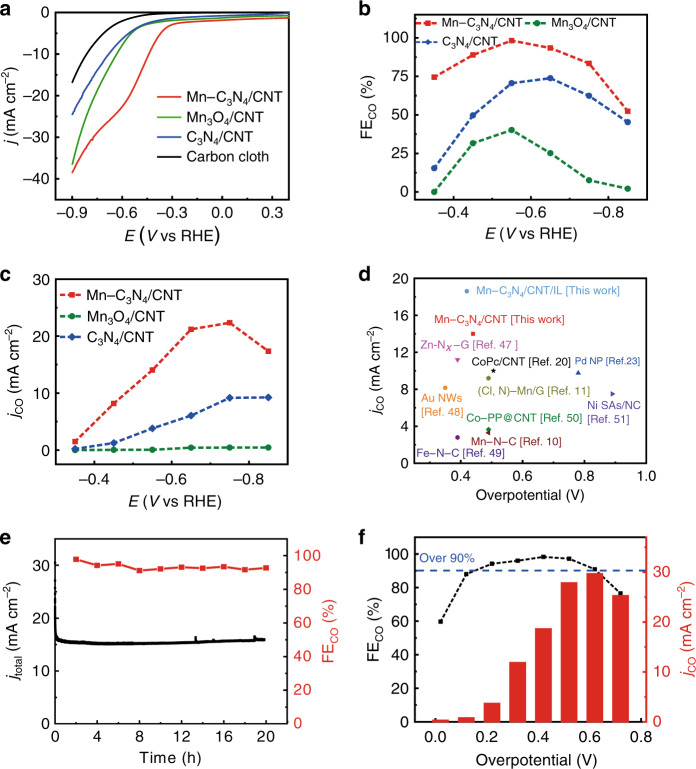
The CO_2_RR performance over Mn–C_3_N_4_/CNT, C_3_N_4_/CNT, and Mn_3_O_4_/CNT. **a** LSV curves in the CO_2_-saturated KHCO_3_ electrolyte (pH: 7.33, temperature: 25 ± 3 °C, without stirring). **b** FE_CO_ and **c** *j*_CO_ at different applied potentials. **d** *j*_CO_ of Mn–C_3_N_4_/CNT at the maximum CO FE compared with those of most state-of-the-art catalysts^10,11,20,23,47–51^. **e** Long-term durability of Mn–C_3_N_4_/CNT operated at −0.55 V for 20 h. **f** FE_CO_ and *j*_CO_ of Mn–C_3_N_4_/CNT at different applied potentials in the CO_2_-saturated [Bmim]BF_4_/CH_3_CN-H_2_O electrolyte.

Inspired by the Mn–N_3_ site in Mn–C_3_N_4_/CNT, its CO_2_RR performance was evaluated in a CO_2_-saturated 0.5 M KHCO_3_ electrolyte by linear sweep voltammetry (LSV). For comparison, C_3_N_4_/CNT, Mn_3_O_4_/CNT and carbon cloth were also measured. Mn–C_3_N_4_/CNT exhibits a more positive onset potential and higher current density than those of other catalysts (Fig. 3a). A cathodic peak appears at approximately −0.5 V (vs. the reversible hydrogen electrode (vs. RHE); all potentials are referenced to RHE) in the LSV curve of Mn–C_3_N_4_/CNT, and the peak current increases linearly with scan rate (Supplementary Fig. 8), which indicates that the peak originates from the CO_2_ mass transfer-controlled electrolysis process^11,19^. Furthermore, the LSV curve of Mn–C_3_N_4_/CNT exhibits the absence of the cathodic peak and the low current density in the N_2_-saturated electrolyte (Supplementary Fig. 9), suggesting its high CO_2_RR activity. The electrolysis tests were performed in an H-type cell to evaluate the selectivity of different catalysts for CO_2_RR. CO and H_2_ were the only gas products in the gas phase and no liquid product was detected. Mn–C_3_N_4_/CNT shows outstanding performance (Fig. 3b), and the CO FE reaches a maximum of 98.8% at −0.55 V, while C_3_N_4_/CNT and Mn_3_O_4_/CNT only show maximum CO FE of 70.6% and 40.2%, respectively. The H_2_ FE of Mn–C_3_N_4_/CNT is much lower than that of C_3_N_4_/CNT and Mn_3_O_4_/CNT at all applied potentials (Supplementary Fig. 10). Combining the total current density and the corresponding CO FE, *j*_CO_ was obtained under different applied potentials. Mn–C_3_N_4_/CNT exhibits a *j*_CO_ of 14.0 mA cm^−2^ at −0.55 V and the highest *j*_CO_ of 22.4 mA cm^−2^ is obtained at −0.75 V (Fig. 3c); these values are much higher than those of C_3_N_4_/CNT and Mn_3_O_4_/CNT (<5 mA cm^−2^). Comparing the performance of various catalysts reported in the literature, it can be concluded that Mn–C_3_N_4_/CNT outperforms all reported Mn SACs and is one of the best reported electrocatalysts for CO_2_ conversion to CO (Fig. 3d, Supplementary Table 2)^20–23^. To further verify the important role of Mn–N_3_ in the reaction, we adopted the method reported in the literature to destroy the structure through an annealing process at 1273 K in a N_2_ atmosphere (with the product denoted as Mn–NC/CNT)^24,25^. Both the CO FE and *j*_CO_ of Mn–NC/CNT decrease evidently compared with those of Mn–C_3_N_4_/CNT (Supplementary Fig. 11), confirming the active site role of Mn–N_3_. To test the stability of the catalyst, a 20 h electrolysis experiment was performed on Mn–C_3_N_4_/CNT, and the CO FE and current density did not obviously decay (Fig. 3e). Additionally, no noticeable change can be observed in the morphology of Mn–C_3_N_4_/CNT, and the atomic contents of C–N–C (29.9%), Mn–N (17.5%), N–(C)_3_ (39.8%), and N–H (12.7%) fall into the statistical region of the atomic content of N moieties for pristine Mn–C_3_N_4_/CNT after long-term electrolysis (Supplementary Fig. 12). All of these results indicate the excellent stability of Mn–C_3_N_4_/CNT for the CO_2_RR. A large electrochemical active surface area (ECSA) can contribute to the activity of catalyst. The measured double-layer capacitance of catalysts is displayed in Supplementary Figs. 13 and 14, in which the slope could be a reference for the ECSA. Mn–C_3_N_4_/CNT and C_3_N_4_/CNT share a similar ECSA, which is much larger than that of Mn_3_O_4_/CNT, suggesting that the g-C_3_N_4_ enveloped on the CNTs surface can increase the ECSA, but it is not the key reason for the final performance of Mn–C_3_N_4_/CNT. Nyquist plots were obtained at the open-circuit potential to investigate kinetic reactions on the electrode/electrolyte interface (Supplementary Fig. 15) and the data were fitted using a simple equivalent circuit R(CR)W. The results show that Mn–C_3_N_4_/CNT undergoes a faster interfacial charge-transfer process during the CO_2_ reaction process than the other catalysts, which could improve the reaction activity.

Compared with aqueous electrolytes, IL electrolytes have many unique physicochemical properties, such as a wide electrochemical potential window, large absorption capacity of CO_2_, and high intrinsic ionic conductivity, which are beneficial to the CO_2_RR^26–28^. Therefore, the activity of Mn–C_3_N_4_/CNT was tested in a CO_2_-saturated IL electrolyte (1-butyl-3-methylimidazolium tetrafluoroborate ([Bmim]BF_4_)/acetonitrile (CH_3_CN)–H_2_O)^29,30^. Since the reference electrode (Ag/Ag^+^ electrode) used for the organic electrolyte is different from that (Ag/AgCl electrode) for aqueous electrolyte and the equilibrium potential for CO is −1.68 V vs. Ag/Ag^+^ in the IL electrolyte (Supplementary Fig. 16)^31,32^, the overpotential is adopted for the following discussion. Figure 3f shows that the CO FE of Mn–C_3_N_4_/CNT maintains over 90% in a wide overpotential range from 0.22 to 0.62 V and reaches a maximum of 98.3% at 0.42 V overpotential. Meanwhile, Mn–C_3_N_4_/CNT exhibits a *j*_CO_ of 18.6 mA cm^−2^ at the maximum CO FE. The highest *j*_CO_ value of 29.7 mA cm^−2^ is obtained at an overpotential of 0.62 V, which is higher than that of Mn–C_3_N_4_/CNT in the KHCO_3_ electrolyte. ^13^CO_2_ was also used as the feedstock to carry out the electrolysis test in the IL, electrolyte and it was confirmed that CO is the conversion product of CO_2_ (Supplementary Fig. 17). The enhanced performance of Mn–C_3_N_4_/CNT in the IL electrolyte is probably based on the following mechanism. First, the CO_2_ solubility of [Bmim][BF_4_] is much higher than that in aqueous solution, which alleviates the resistance of mass transfer on the CO_2_RR^33^. Second, it has been reported that the imidazolium cation of IL can electrostatically be arranged at the cathode to favor CO_2_ adsorption via [Bmim-CO_2_]^+^, which could help to lower the activation energy for the CO_2_ reduction process^27,34^. Therefore, it is an efficient strategy to enhance the performance of catalysts by integration with an IL electrolyte, especially in terms of the current density and the overpotential.

## Discussion

**Fig. 4:**
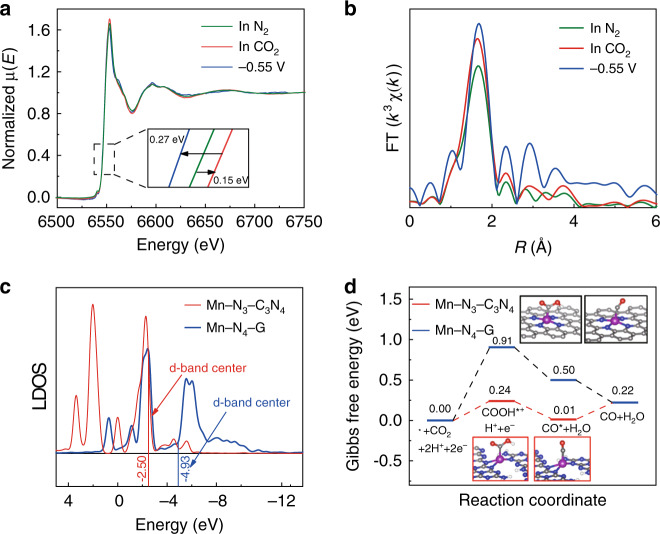
In situ XAS experiments and DFT calculations. **a**, **b** XANES and EXAFS spectra at the Mn K-edge of Mn–C_3_N_4_/CNT under various conditions. **c** Local density of states (LDOS) of Mn in Mn–N_3_–C_3_N_4_ and Mn–N_4_-G. **d** Calculated Gibbs free energy diagrams for CO_2_RR over Mn–N_3_–C_3_N_4_ and Mn–N_4_-G.

The results mentioned above demonstrate that Mn–C_3_N_4_/CNT has excellent CO_2_RR activity. Therefore, in situ XAS analysis and DFT calculations were performed to shed light on the process of CO_2_ reduction over Mn–C_3_N_4_/CNT. The Mn K-edge XANES and EXAFS spectra of Mn–C_3_N_4_/CNT were recorded under in situ conditions. As shown by the XANES spectra (Fig. 4a), the Mn K-edge of Mn–C_3_N_4_/CNT increased by 0.15 eV in the CO_2_-saturated electrolyte under the open-circuit voltage compared with that in the N_2_-saturated electrolyte. This result could be attributed to the increase in the Mn oxidation state owing to the redistribution of electrons after CO_2_ adsorption on the Mn site, transferring from the Mn site to the carbon 2*p* orbital in CO_2_ to form a CO_2_^•−^ species^35^. When a potential of −0.55 V was applied on the electrode in the CO_2_-saturated electrolyte, a CO_2_ electroreduction reaction occurred, and the Mn K-edge of Mn–C_3_N_4_/CNT shifted back to lower energy, which suggests that the Mn valence state returns to the low valence state after one cycle of CO_2_ reduction^36^. In the FT EXAFS spectra (Fig. 4b), the intensity of the main peak at ~1.7 Å increased slightly in the CO_2_-saturated electrolyte compared with that in the N_2_-saturated electrolyte, which can be attributed to the formation of a C–Mn^37^ bond when the Mn site interacted with CO_2_. The main peak shifted to a longer length (~1.76 Å) when applying voltage to the electrode, indicating the lengthening of the Mn–N bond due to the redistribution of electrons among Mn, N, and C (from adsorbed CO_2_). The results from the in situ XAS spectra indicate that the Mn–N_3_ site in Mn–C_3_N_4_/CNT is the active site for the CO_2_RR, on which CO_2_ is adsorbed, activated, and converted.

The coordination number of Mn in Mn–C_3_N_4_/CNT is 3.2, which is slightly different from that of the Mn–N_3_ moiety. In general, it is accepted by researchers to use the integral value of the best-fitted coordination number to represent the structure of the catalyst active site in DFT calculations^38,39^, because there is an error bound while using the data from EXAFS to fit the coordination number. Therefore, a coordination number of three (Mn–N_3_) is adopted as the catalyst structure for building a DFT model. The catalyst structure is optimized according to the literature^18^, which indicated that a single atom of Mn interacting with the edge of g-C_3_N_4_ is the optimal structure. On this basis, the structure of the catalyst is further optimized and the results indicate that the Mn atom interacting with the edge of g-C_3_N_4_ via three bonds (Mn–N_3_–C_3_N_4_) is most stable. Moreover, the coordination number and the average Mn–N bond length (2.11 Å) of Mn–N_3_–C_3_N_4_ are basically consistent with the EXAFS results. Therefore, Mn–N_3_–C_3_N_4_ is confirmed as the structure of Mn–C_3_N_4_/CNT, which is obviously different from that of reported Mn SACs (Mn–N_4_ moiety embedded in graphene, Mn–N_4_-G)^10,13^ (Supplementary Figs. 18 and 19, Supplementary Table 3, and Supplementary Note 1). The positions of the *d*-band centers of Mn–N_3_–C_3_N_4_ and Mn–N_4_-G were calculated to investigate their electronic structures. Figure 4c shows that the position of the *d*-band center for Mn–N_3_–C_3_N_4_ is closer to the Fermi level than that of Mn–N_4_–G, indicating Mn–N_3_–C_3_N_4_ is more favorable to the binding and activation of CO_2_. The above results suggest that the electronic structures of Mn–N_3_–C_3_N_4_ and Mn–N_4_–G are different, which may lead to different catalytic mechanisms. Thus, DFT calculations were performed to investigate the mechanisms of CO_2_RR. The CO_2_ electroreduction to CO involves two electrons and two protons: (I) CO_2_ + _*_ + H^+^ + e^−^ → ^*^COOH; (II) ^*^COOH + H^+^ + e^−^ → ^*^CO + H_2_O; and (III) ^*^CO → CO + _*_ (* represents the active site of catalyst). The structures of the COOH* and CO* reaction intermediates on the catalysts are displayed in Supplementary Figs. 20 and 21, it can be seen that the Mn atoms in Mn–N_3_–C_3_N_4_ are distorted out of the g-C_3_N_4_ plane, and the average Mn–N bond length (2.18 Å) is longer than that of the original Mn–N_3_–C_3_N_4_ (2.11 Å) after interacting with COOH^*^, which is in accordance with the in situ XAS spectral results. Moreover, Mn–N_3_–C_3_N_4_ presents a higher adsorption energy of COOH^*^ (Δ*E*_ads_ = −1.998 eV) than Mn–N_4_–G (Δ*E*_ads_ = −1.367 eV), indicating that COOH^*^ is more stable on Mn–N_3_–C_3_N_4_ than Mn–N_4_–G (Supplementary Table 4). The free energy profiles of CO_2_ electroreduction to CO are shown in Fig. 4d. The potential-determining steps of Mn–N_3_–C_3_N_4_ and Mn–N_4_–G are both the first-step reduction of CO_2_ to form the COOH^*^ intermediate, corresponding to free energy increases of 0.24 and 0.91 eV, respectively. Clearly, Mn–N_3_–C_3_N_4_ has much a lower Gibbs free energy for COOH^*^ formation. The results of the DFT calculations confirm that Mn–N_3_–C_3_N_4_ is an efficient site for CO_2_ conversion.

In summary, we fabricated a Mn SAC with a Mn–N_3_ active site and applied it to the CO_2_RR. The synthesized Mn SAC exhibited a 98.8% CO FE with a high *j*_CO_ of 14 mA cm^−2^ at an overpotential of 0.44 V in aqueous electrolyte. When an IL is used as the electrolyte, higher *j*_CO_ of 18.6 and 29.7 mA cm^−2^ are obtained at an overpotential of 0.42 and 0.62 V, respectively. In situ XAS spectra and DFT calculations indicate that the Mn–N_3_ site is the active center, on which CO_2_ is more easily adsorbed and the free energy barrier of key intermediate formation is greatly decreased. It can be anticipated that Mn SACs may also exhibit excellent performance in other electrochemical reactions by changing the supporting materials to form exclusive active sites.

## Methods

### Materials

Manganous acetate (Mn(COOH)_2_·4H_2_O) (99.99%) and DCD (>98%) were purchased from Shanghai Aladdin Biochemical Tech. Co., Ltd. Multiwalled carbon nanotubes (MWCNTs) were provided by XFNANO. Carbon cloth (HCP331P, 19 × 19 cm), Nafion D-521 dispersion (5% w/w in water and 1-propanol, ≥0.92 meg/g exchange capacity), and Nafion N-117 membrane (0.180 mm thick, ≥0.90 meg/g exchange capacity) were purchased from Shanghai Hesen Electric Co., Ltd. 1-butyl-3-methylimidazolium tetrafluoroborate ([Bmim]BF_4_, purity>99%) was purchased from Shanghai Chengjie Co., Ltd. The IL was purified and dried before use. Sulfuric acid (AR grade) was purchased from Beijing Chemical Company. All aqueous solutions were prepared with Milli-Q water.

### Surface modification of MWCNTs

Hydrophilic functional groups were produced on the surface of the MWCNTs by oxidation treatment. First, 200 mg of pristine MWCNTs was dispersed in 50 mL of mixed acid (3:1 v/v solution of sulfuric acid and nitric acid) with the assistance of sonication and then stirred at room temperature for 24 h. After repeatedly washing with water, the treated MWCNTs were stirred with 25 mL of 5 M nitric acid at room temperature for 24 h to remove metal impurities, sequentially washed with water and finally vacuum dried.

### Synthesis of Mn–C_3_N_4_/CNT

First, 25 mg of treated MWCNTs, 50 mg of DCD and 24 mg of Mn(COOH)_2_·4H_2_O were sonicated in 100 mL of water and then stirred at 323 K for 10 h. The mixture was dried using lyophilization and annealed at 873 K for 1 h under a nitrogen atmosphere. To obtain the final product of Mn–C_3_N_4_/CNT, the as-annealed powders were further washed with 1 M HCl followed by water and finally were subjected to vacuum drying.

### Synthesis of Mn_3_O_4_/CNT and C_3_N_4_/CNT

Mn_3_O_4_ supported on CNTs was prepared by the same procedure as Mn–C_3_N_4_/CNT except for the omissions of DCD and HCl treatment. C_3_N_4_/CNT was prepared by the same procedure as Mn–C_3_N_4_/CNT except for the omission of metal salt.

### Synthesis of Mn–C_3_N_4_ and pure g-C_3_N_4_

Mn–C_3_N_4_ was synthesized by the same procedure as Mn–C_3_N_4_/CNT except for the omission of MWCNTs. Pure g-C_3_N_4_ was prepared by the same procedure as Mn–C_3_N_4_/CNT except for the omissions of MWCNTs and metal salt.

### Physical and chemical characterizations

Powder XRD measurements were recorded with a Rigaku Smartlab diffractometer with Cu Kα radiation (*λ* = 1.5418 Å) operated at 45 kV and an emission of 50 mA. The scattering range of 2*θ* was from 5° to 90°, with a scanning rate of 15 min^−1^. SEM was performed using a Hitachi SU8020 electron microscopy operated at 5 kV. TEM was performed on a JEOL JEM-2100 system. High-angle annular dark-field scanning transmission electron microscopy (HAADF-STEM) characterization and corresponding energy-dispersive spectroscopy (EDS) were conducted on a JEOL JEM-ARF200F TEM/STEM system with a spherical aberration corrector. XPS was performed by a Thermo Fisher Scientific ESCALAB 250Xi using an Al Kα (1486.6 eV) X-ray source under a pressure of 3 × 10^−7^ mbar, and the binding energy was referenced to the C 1*s* peak at 284.8 eV. The N_2_ adsorption–desorption isotherms of catalysts were recorded at 77 K on a Quantachrome Instrument NOVA 2000, and the specific surface area and pore size distribution were determined by the Brunauer–Emmett–Teller (BET) method and Barrett–Joyner–Halenda (BJH) model, respectively. The X-ray absorption fine structure spectra were collected at the 1W1B station in the Beijing Synchrotron Radiation Facility (BSRF). The storage rings of the BSRF were operated at 2.5 GeV with an average current of 250 mA. Using a Si (111) double-crystal monochromator, the data collection was carried out in fluorescence mode using an ionization chamber. All spectra were collected under ambient conditions, and XAFS data were processed according to standard procedures using the ATHENA module implemented in the IFEFFIT software packages. The *k*^3^-weighted EXAFS spectra were obtained by subtracting the post-edge background from the overall absorption and then normalized with respect to the edge-jump step. Subsequently, the *k*^3^-weighted *χ*(*k*) data of the Mn K-edge were FT to real (R) space using hanging windows (dk = 1.0 Å^−1^) to separate the EXAFS contributions from different coordination shells. To obtain the quantitative structural parameters around central atoms, least-squares curve parameter fitting was performed using the ARTEMIS module of IFEFFIT software packages^40–42^.

### Electrochemical test

Fabrication of the working electrode: 1.0 mg of the catalyst was suspended in 240 μL of isopropanol and 40 μL of Nafion D-521 with ultrasound assistance to form a homogeneous ink. Then, the ink was dropped onto a carbon cloth (1 × 1 cm^2^) surface by a micropipette and finally dried at room temperature. CO_2_RR was conducted in a typical H-type electrochemical cell separated by a Nafion 117 membrane with a three-electrode system. A Ag/AgCl or Ag/Ag^+^ electrode and platinum gauze were used as the reference electrode and counter electrode, respectively. The working and reference electrodes were placed in the cathode chamber with a 0.5 M KHCO_3_ solution or [Bmim][BF_4_]/CH_3_CN–H_2_O mixture solution containing 30 wt% [Bmim][BF_4_], 65 wt% CH_3_CN, and 5 wt% H_2_O as the electrolyte, while the counter electrode was placed in the anode chamber with a 0.1 M H_2_SO_4_ solution as the electrolyte. Before the experiment, the electrolyte in the cathode chamber was bubbled with N_2_ or CO_2_ (30 mL min^−1^) for at least 30 min to form the N_2_-saturated or CO_2_-saturated solution, respectively, and magnetic stirring (240 r min^−1^) was applied to the electrolyte during the experiment for better mixing. LSV measurements were recorded at a scan rate of 20 mV s^−1^. ECSA referred to the cyclic voltammogram results under the potential windows of 0.5 V ~ 0.6 V with different scan rates of 5, 10, 20, 40 and 60 mV s^−1^. For the FE analysis, the gas products were collected into a sampling bag. After electrolysis for 1 h, the products were analyzed and quantified by a gas chromatography (GC, Agilent 6820) system equipped with a thermal conductivity detector (TCD) and a flame ionization detector (FID) using nitrogen as the carrier gas. ^1^H nuclear magnetic resonance (NMR) spectroscopy (Bruker Advance III HD 600) was employed to confirm and quantify the liquid products with phenol as an internal standard in DMSO-d_6_. The quantification of gas and liquid products was determined by comparison with the standard curves. On the basis of GC and ^1^H NMR analysis, the respective current density and FE of the products were calculated. The CO FE were calculated by the following formula:

1 $${\mathrm{{FE}}} = \frac{{NnF}}{Q} \times 100$$

where *N* represents the number of electrons transferred for product formation, which is 2 for CO, *n* is the total moles number of CO measured by GC, *F* is the Faraday constant (96,485 C mol^−1^) and *Q* is the amount of cumulative charge recorded by the electrochemical workstation.

### In situ XAS

A homemade electrochemical cell was employed for in situ XAS experiments under the sensitive fluorescence model. The cell was filled with the electrolyte, and a Ag/AgCl electrode and platinum gauze were used as the reference electrode and counter electrode, respectively. There is a single circular hole of 1.0 cm in diameter on the wall of the cell. The working electrode contacted with a slip of copper tape was placed to the exterior of the wall to cover the hole and Kapton (polyimide) tape was used to seal the cell. In situ XAS spectra were obtained at the open-circuit potential in N_2_ or CO_2_-saturated electrolyte and an applied potential of −0.55 V in the CO_2_-saturated electrolyte.

### Computational details for calculations

We carried out first-principle DFT calculations on the CO_2_RR activity of Mn–N_3_–C_3_N_4_ and Mn–N_4_–G by the projector-augmented wave (PAW) method-based Vienna ab initio simulation package (VASP)^43,44^. The functional of Perdew, Burke, and Ernzerhof (PBE) with generalized gradient approximation (GGA) was considered for the electron exchange-correlation^45^. The cutoff energy for the plane-wave basis set was set as 450 eV. The vacuum layer was set to be more than 12 Å to preclude coupling between periodic supercells. A Monkhorst–Pack *k*-point mesh of 5 × 5 × 1 grid was used to sample the Brillouin zone. The convergence criteria were set to be <10^−5^ eV in total energy and 0.01 eV Å^−1^ in force. The adsorption energy of the COOH* and CO* intermediates were defined as the energy difference between the adsorbed complex and the sum of isolated Mn–N_3_–C_3_N_4_/Mn–N_4_–G) catalyst and the isolated COOH^*^(CO^*^) group. For the electroreduction process, the model of a computational hydrogen electrode was used to calculate the energy of the proton–electron pair^46^. The Gibbs free energy (Δ*G*) for each reaction process is calculated with the following equation:

2 $$\Delta G = \Delta E + \Delta E_{{\mathrm{ZPE}}} - T\Delta S$$

where Δ*E*, Δ*E*_ZPE_ and Δ*S*, denote the changes in the DFT electronic energy, the zero-point energy, and the entropy at 300 K, respectively. For the vibrational entropy calculations, only the adsorbed species were allowed to move, while the catalytic substrate such as Mn–N_3_–C_3_N_4_ or Mn–N_4_–G was fixed.

## Supplementary information

Supplementary Information

## Data Availability

The data supporting the findings of this study are available within the article and its Supplementary information files. All data is available from the authors upon reasonable request.

## Code availability

All code supporting the findings of this study are available from the corresponding author on request.
