# Supplementary material for: A Mn-N3 single-atom catalyst embedded in graphitic carbon nitride for efficient CO2 electroreduction
Source: Nat Commun. 2020 Aug 28;11:4341. doi: 10.1038/s41467-020-18143-y (PMC7455739; doi:10.1038/s41467-020-18143-y)
Supplement: Supplementary file 1 — Supplementary Information [file 41467_2020_18143_MOESM1_ESM.pdf]

A Mn-N_3 single-atom catalyst embedded in graphitic carbon nitride for efficient CO_2 electroreduction

Jiaqi Feng et al.

Supplementary Figures

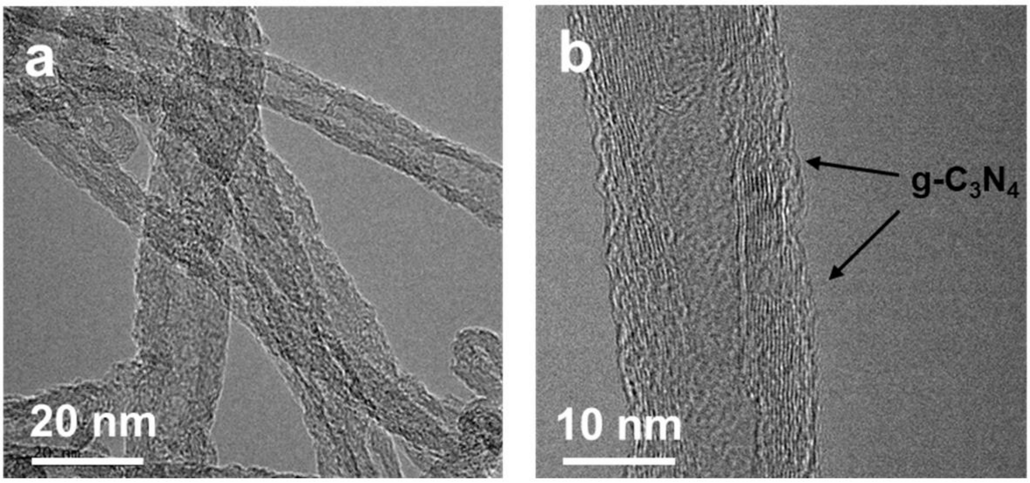

Supplementary Figure 1 | Characterizations of C_3N_4/CNT. (a) Large-field view and (b) magnified view of TEM images of C_3N_4/CNT.

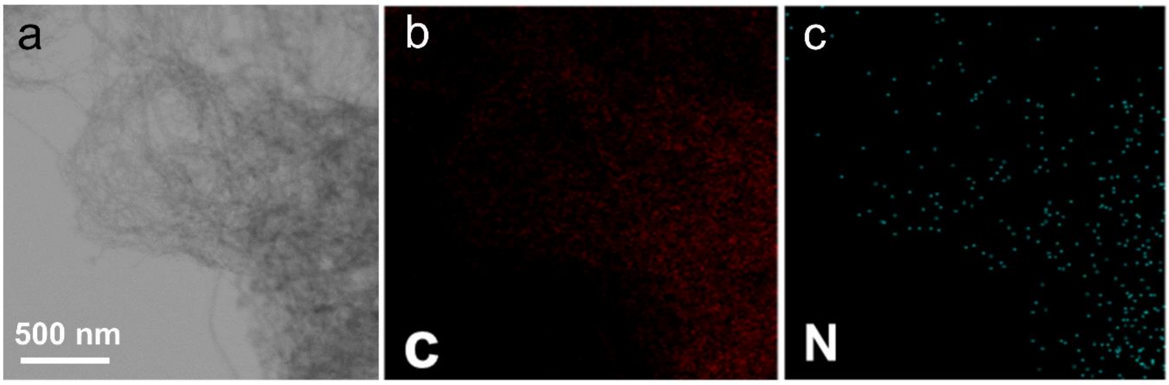

Supplementary Figure 2 | EDS mapping images of C_3N_4/CNT. (a) STEM, (b) C element and (c) N element images

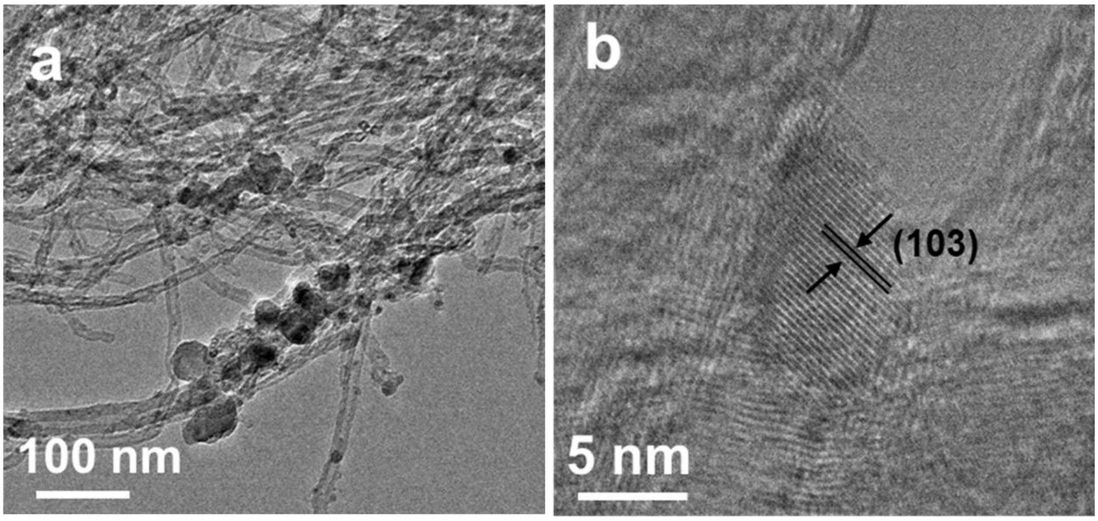

Supplementary Figure 3 | Characterizations of Mn/CNT. (a) TEM and (b) HRTEM images of Mn/CNT.

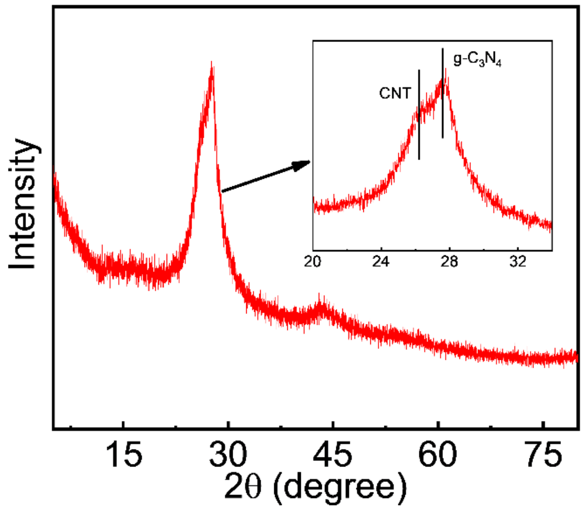

Supplementary Figure 4 | XRD pattern of Mn-C_3N_4/CNT. The amount of DCD used in the synthesis process is 400 mg.

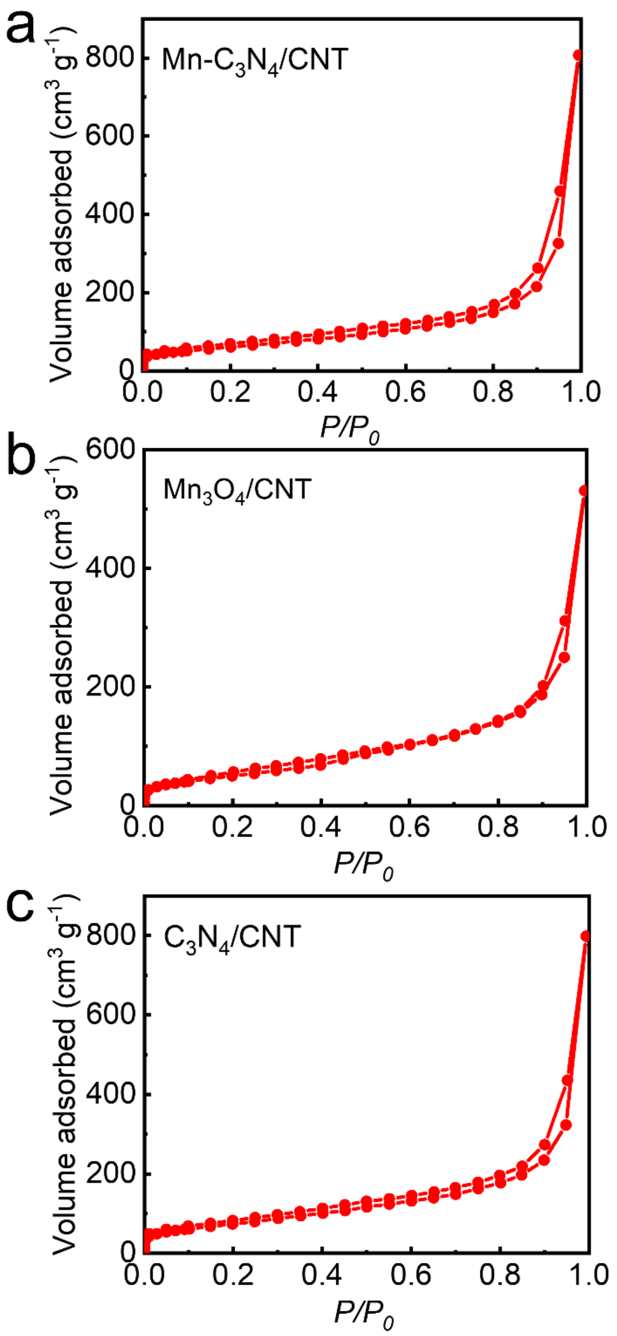

Supplementary Figure 5 | N_2 adsorption-desorption isotherms. (a) Mn-C_3N_4/CNT, (b) Mn_3O_4/CNT and (c) C_3N_4/CNT.

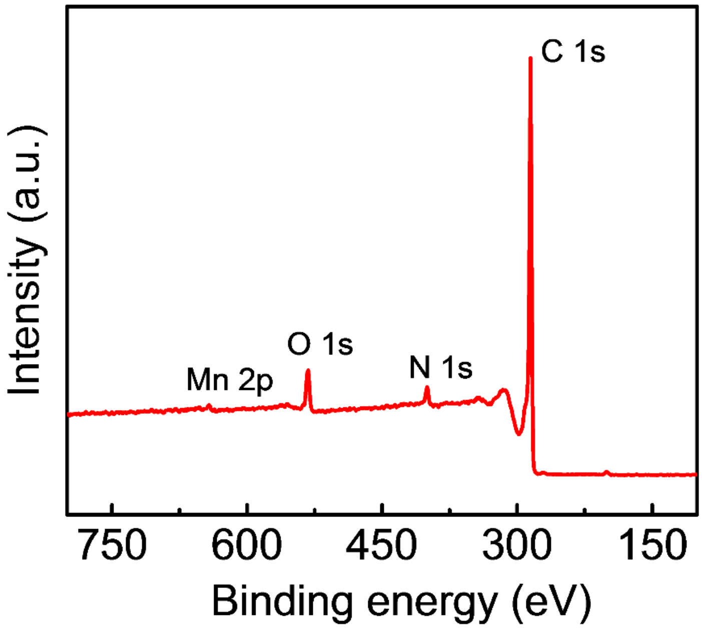

Supplementary Figure 6 | Full XPS spectrum of Mn-C_3N_4/CNT.

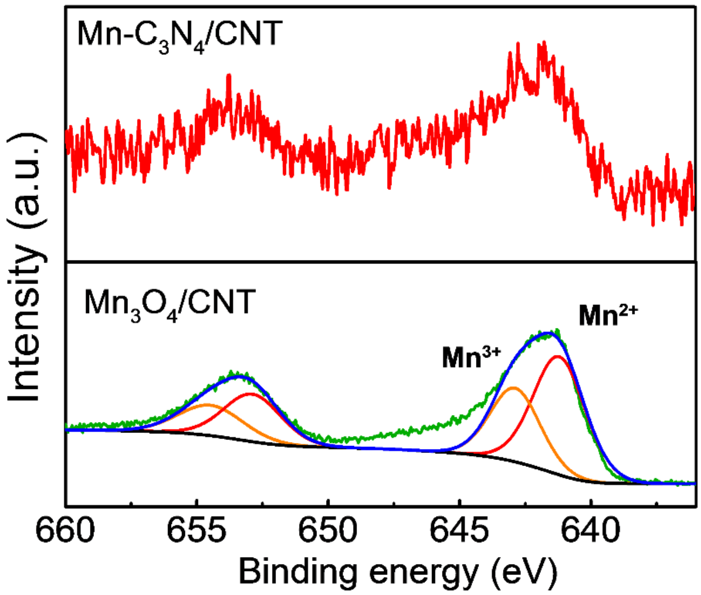

Supplementary Figure 7 | High-resolution Mn 2p XPS spectra of Mn-C_3N_4/CNT and Mn_3O_4/CNT.

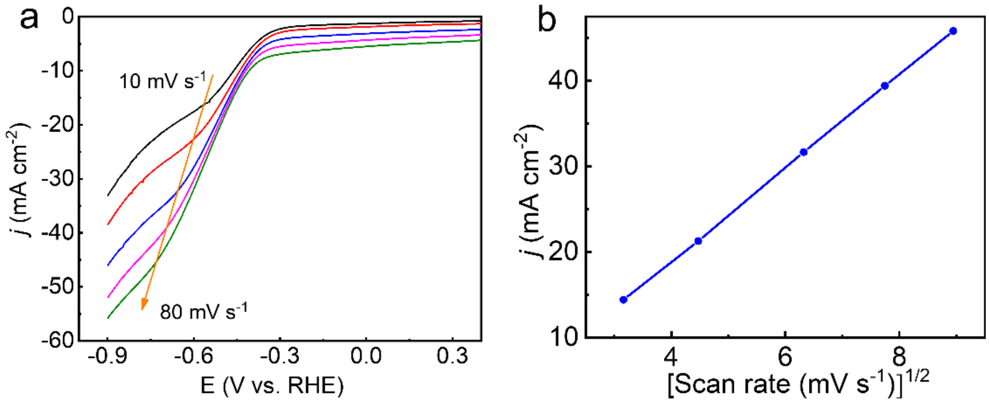

Supplementary Figure 8 | Electrochemical CO_2RR performance on Mn-C_3N_4/CNT. (a) Scan rate dependent LSV response and (b) peak current density of Mn-C_3N_4/CNT *vs*. scan rate$^{1/2}$.

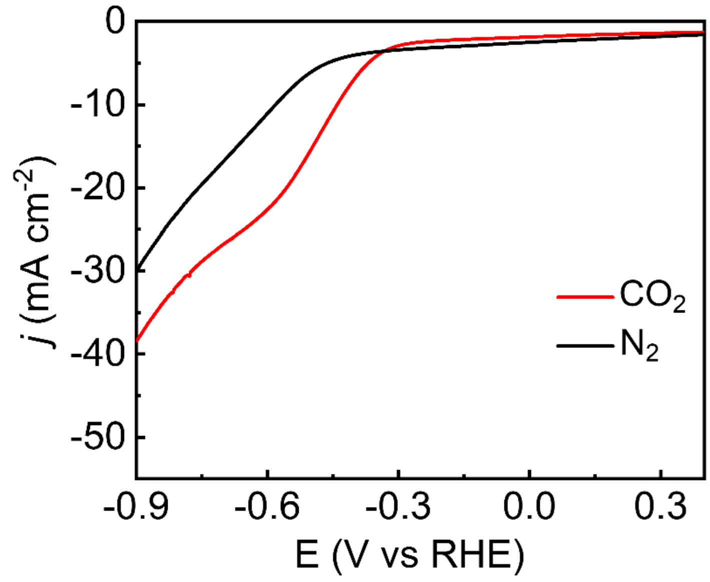

Supplementary Figure 9 | LSV curves for Mn-C_3N_4/CNT in CO_2- and N_2-saturated 0.5 M $KHCO_3$ solution.

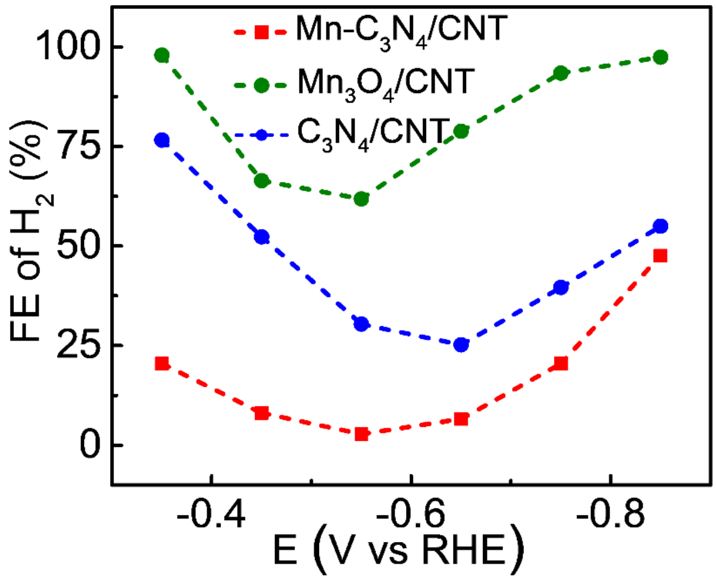

Supplementary Figure 10 | FE of H_2 at different applied potentials in the CO_2-saturated $KHCO_3$ electrolyte.

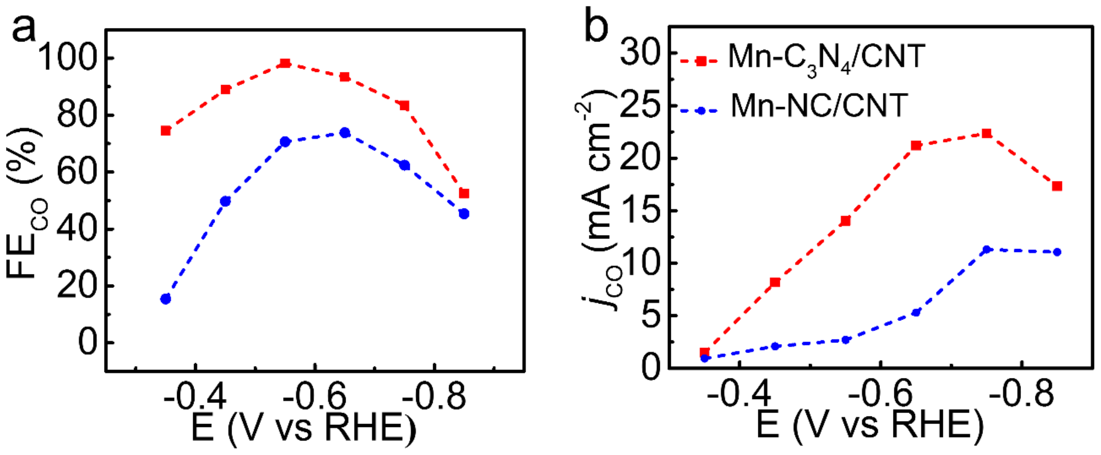

Supplementary Figure 11 | The CO_2RR performance of Mn-C_3N_4/CNT and Mn-NC/CNT in the CO_2-saturated $KHCO_3$ electrolyte. (a) Potential-dependent FE_{CO} and (b) CO partial current density.

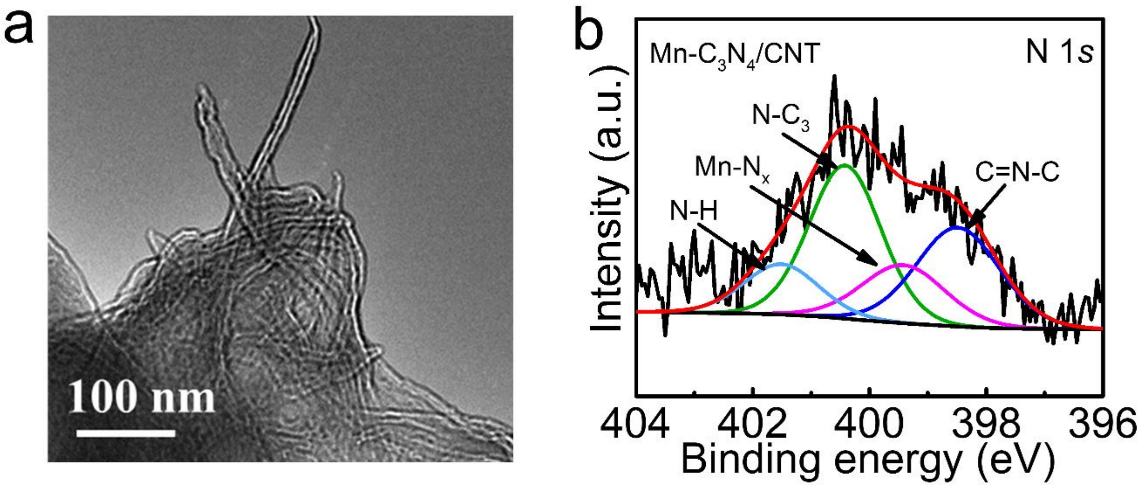

Supplementary Figure 12 | The characterizations of Mn-C_3N_4/CNT after long-term electrolysis. (a) TEM image and the CNT agglomeration is caused by the addition Nafion D-521 solution in the process of the working electrode fabrication. (b) High-resolution N 1*s* XPS spectrum of Mn-C_3N_4/CNT.

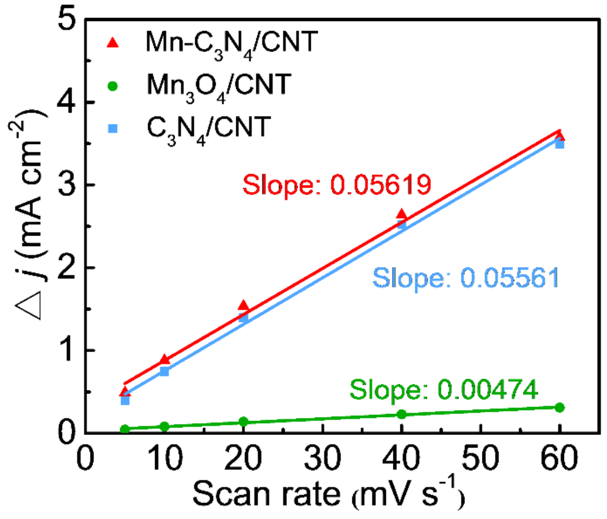

Supplementary Figure 13 | Charging current density differences plotted against scan rates.

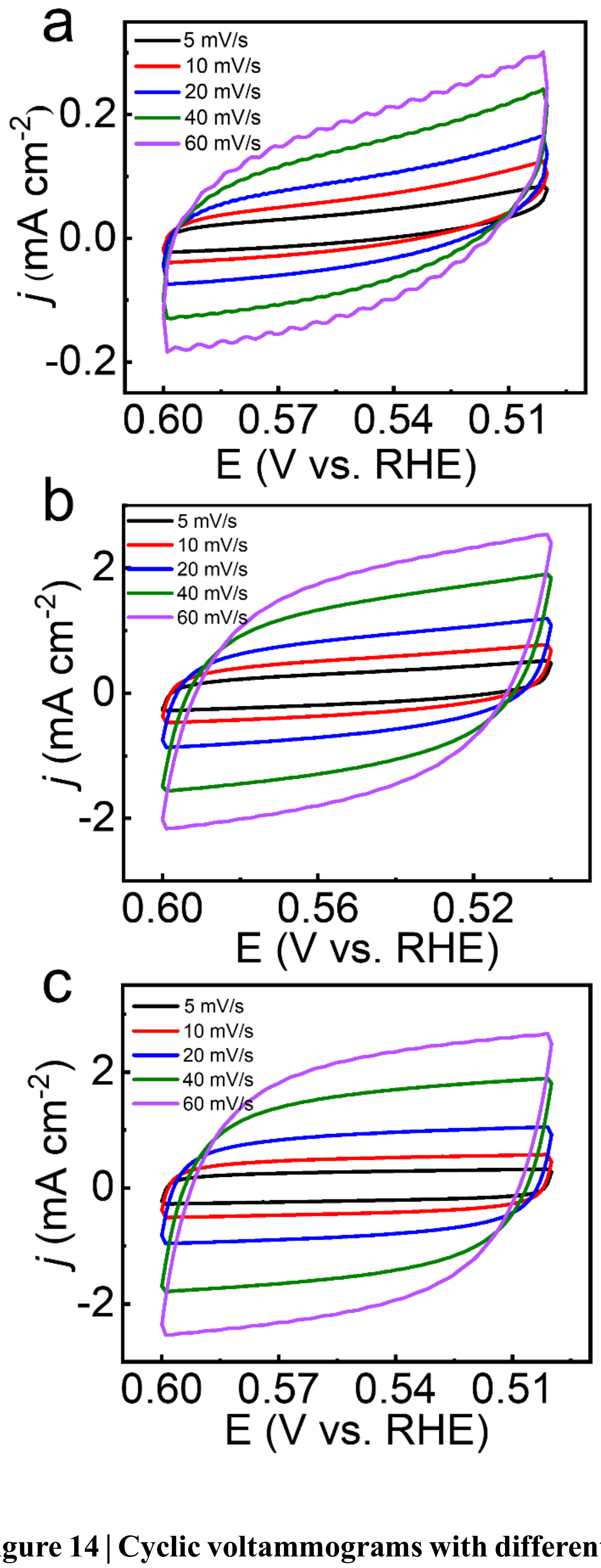

Supplementary Figure 14 | Cyclic voltammograms with different scan rate. (a) Mn-C_3N_4/CNT, (b) Mn_3O_4/CNT, (c) C_3N_4/CNT.

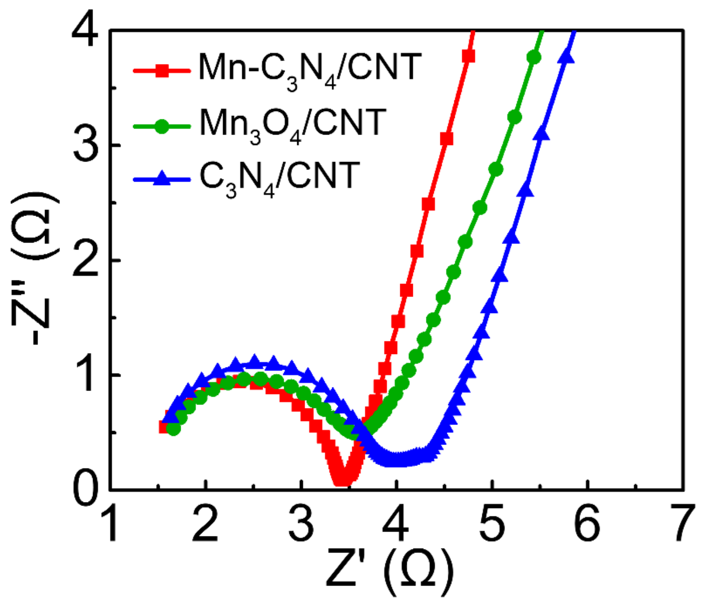

Supplementary Figure 15 | EIS spectra of different catalysts at the **open circuit potential.**

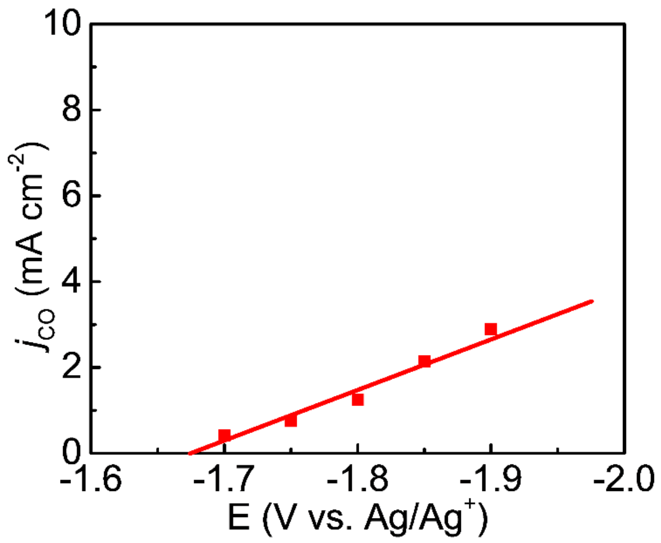

Supplementary Figure 16 | Partial current densities of CO under different potentials. The equilibrium potential can be obtained by extrapolation zero partial current density.

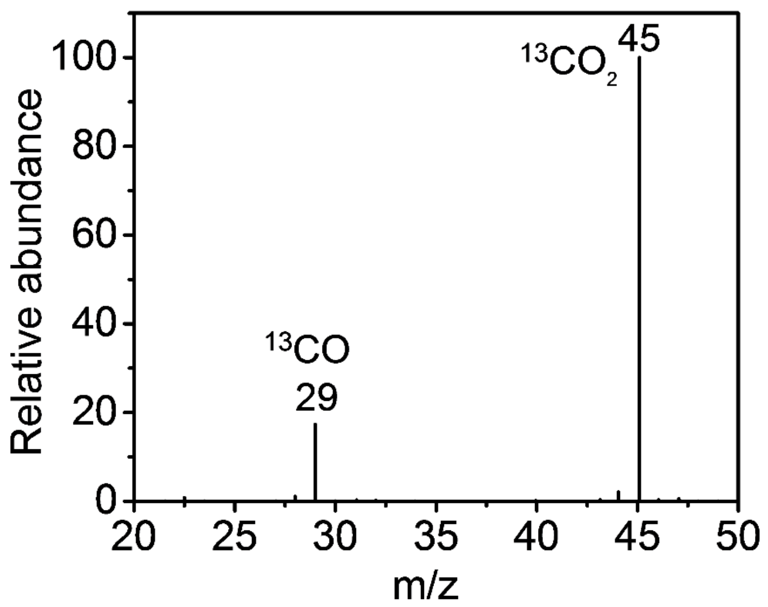

Supplementary Figure 17 | Mass spectrometry signal of CO_2RR using $^{13}CO_2$ as the feedstock.

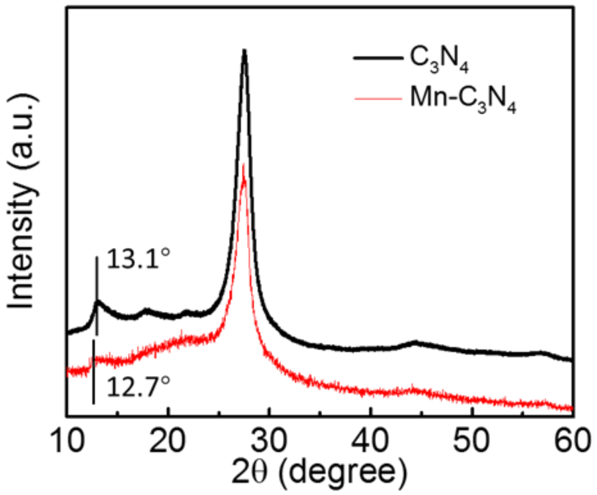

Supplementary Figure 18 | XRD patterns of C_3N_4 and Mn-C_3N_4. In order to investigate the effect of Mn on g-C_3N_4 and exclude the covering of CNT on C_3N_4 peaks, the XRD patterns of the pure g-C_3N_4 and Mn-C_3N_4 were conducted. Two peaks at around 27.3° and 13.1° are attributed to typical g-C_3N_4 structure.[1] The former peak is related to the interplanar stacking of aromatic systems and the latter peak is attributed to the in-plane repeated melon units. Interestingly, the latter peak shifts significantly from 13.1° to 12.7° after doping of Mn in g-C_3N_4 structure, indicating that Mn atoms enlarge the distance among the melon units.[2]

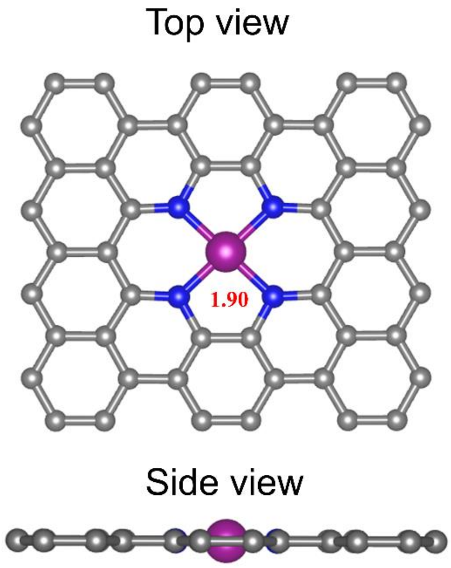

Supplementary Figure 19 | The optimized structures of Mn-N_4 embedded in graphene (Mn-N_4-G). The Mn-N bond lengths in each structure are shown inset (indicated by red), Mn (purple), N (blue), C (gray).

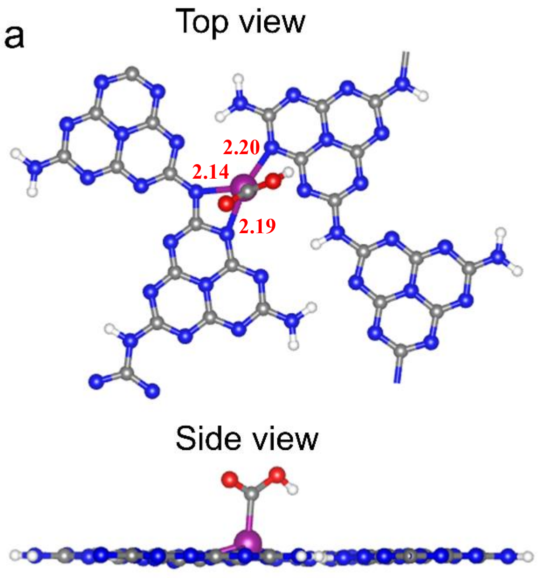

Supplementary Figure 20 | The structures of COOH* intermediates on catalysts. (a) Mn-N_3-C_3N_4; (b) Mn-N_4-G. The Mn-N bond lengths in each structure are shown inset (indicated by red), Mn (purple), N (blue), C (gray), H (white).

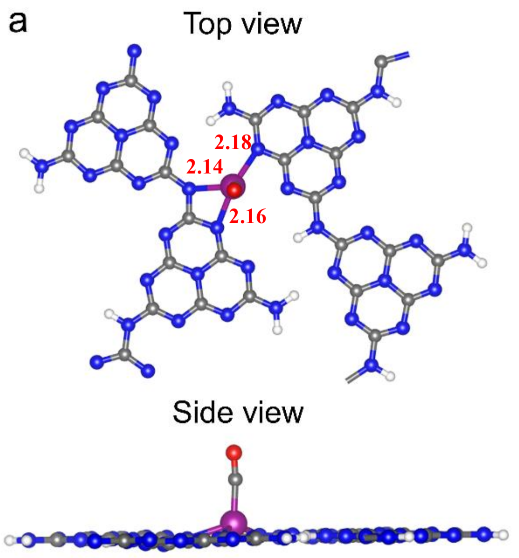

Supplementary Figure 21 | The structures of CO* intermediates on catalysts. (a) Mn-N_3-C_3N_4; (b) Mn-N_4-G. The Mn-N bond lengths in each structure are shown inset (indicated by red), Mn (purple), N (blue), C (gray), H (white).

Supplementary Tables

Supplementary Table 1 | EXAFS fitting parameters at the Mn K-edge for sample.

Sample	Shell	*CN*	R (Å)	σ^2 (Å^2·10^{-3})	ΔE_0 (eV)	R factor (%)
Mn-C_3N_4/CNT	Mn-N	3.2	2.21	6.2	3.3	0.4

CN: coordination numbers; *R*: bond distance; σ^2: Debye-Waller factors (a measure of thermal and static disorder in absorber-scatter distances); ΔE_0: the inner potential correction (the difference between the zero kinetic energy value of the sample and that of the theoretical model). *R* factor: goodness of fit. S_0^2 for Mn-N was set as 0.8, which was obtained from the experimental EXAFS fit of MnPc reference by fixing *CN* as the known crystallographic value and was fixed to all the samples. Error bounds that characterize the structural parameters obtained by EXAFS spectroscopy were estimated as *CN* ± 20%; σ^2 ± 20%; R ± 0.03Å.

Supplementary Table 2 | Performance comparison of various reported CO_2RR electrocatalysts.

Catalysis	Electrolyte	Overpotential (V)	FE_{CO} (%)	j_{CO} (mA cm^{-2})	Ref.
Mn-N-C	0.5M $KHCO_3$	0.44	~40	~0.8	3
Mn-N-C	0.5M $KHCO_3$	0.49	~65	~3.3	4
Mn-N-C	0.5M $KHCO_3$	0.49	~70	~0.7	5
(Cl, N)-Mn/G	0.5M $KHCO_3$	0.49	97	9.2	6
Mn-C_3N_4/CNT	[Bmim]BF_4	0.42 (-2.1 vs. Ag/Ag^+)	98.3	18.6	This work
		0.62 (-2.3 vs. Ag/Ag^+)	90.9	29.7	
	0.5M $KHCO_3$	0.44	98.8	14.0	
		0.64	83.4	22.4	
Ag	[Bmim]BF_4	-2.2 vs. Ag/Ag^+	82.5	2	7
Au foil	[Bmim]BF_4	-2.5 vs. Fc/Fc^+	~90	~6	8
Au nanowires	0.5M $KHCO_3$	0.35	97	8.16	9
Nanoporous Ag	0.5M $KHCO_3$	0.39	92	8.7	10
Pd nanopartical	0.5M $KHCO_3$	0.78	91	9.76	11
ZnN_x-C	0.5M $KHCO_3$	0.32	95	~1	12
		0.51	85	~6	
Zn-N_x-G	0.5M $KHCO_3$	0.39	90.8	11.2	13
CoPc/CNT	0.5M $KHCO_3$	0.52	92	~10	14

Co-PP@CNT	0.5M $NaHCO_3$	0.49	98	3.65	15
Co-N_5/HNPCSs	0.5M $KHCO_3$	0.68	99.3	10.1	16
		0.49	94	~2.5	
COF-367-Co	0.5M $KHCO_3$	0.56	91	3.3	17
CoPc-P4VP	0.5M $KHCO_3$	0.62	89	1.78	18
Ni SAs/N-C	0.5M $KHCO_3$	0.89	71.9	7.5	19
A-Ni-NSG	0.5M $KHCO_3$	0.61	94	22.1	20
SE-Ni Sas@PNG	0.5M $KHCO_3$	0.49	95	~2	21
Fe-N-C	0.5M $KHCO_3$	0.36	93	2.8	22
FePGH	0.5M $KHCO_3$	0.39	96	0.42	23
Fe-N_5	0.1M $KHCO_3$	0.35	96.6	~2.6	24
Bi SAs/NC	0.1M $NaHCO_3$	0.39	97	4	25
		0.64	~72	~8.2	

Supplementary Table 3 | The total energy and structure of optimized Mn-C_3N_4 at different lattice parameters. Mn (purple), N (blue), C (gray), H (white).

Serial number	Lattice parameter (Å)	Total energy (eV)	Structure
I	a = 12.7, b = 16.9	-566.938	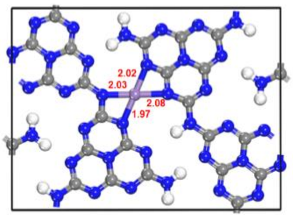
II	a = 12.7, b = 17.2	-566.880	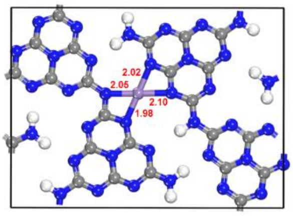
III	a = 12.7, b = 17.5	-566.688	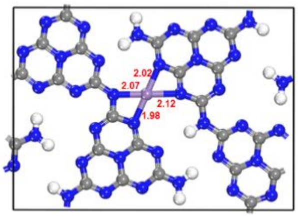
IV	a = 12.7, b = 17.6	-567.133	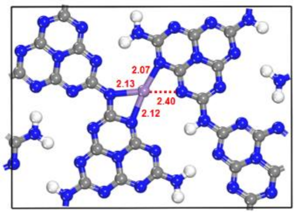
V	a = 12.7, b = 17.7	-567.102	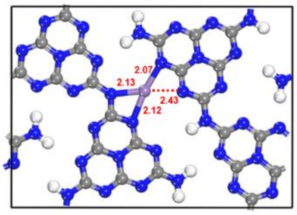
VI	a = 12.7, b = 17.8	-567.067	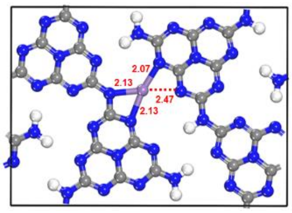

VII	a = 12.7, b = 17.9	-567.008	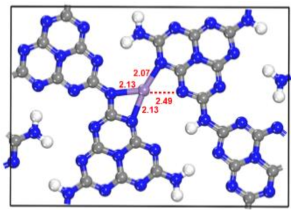
VIII	a = 12.8, b = 17.6	-567.217	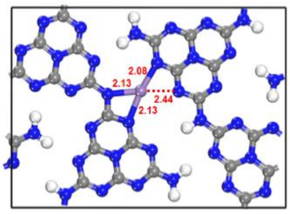
IX	a = 12.9, b = 17.6	-567.216	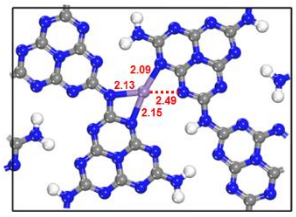
X	a = 13.0, b = 17.6	-567.175	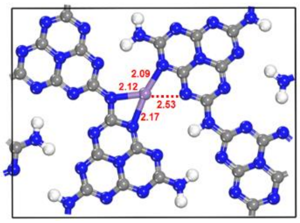

Supplementary Table 4 | The DFT total energies (E_{DFT}), zero-point energies (E_{ZPE}), entropies ($T*S$) multiplied by temperature (T = 300 K), free energies (G), relative free energies (ΔG) at U = 0 *vs.* SHE, and adsorption energies (Eads) of CO_2 reduction intermediates on Mn-N_3-C_3N_4 and Mn-N_4-G.

Catalysts	E_{DFT} (eV)	E_{ZPE} (eV)	$T*S$ (eV)	G (eV)	ΔG (eV)	Eads (eV)
Mn-N_3	-567.217	--	--	-567.217	--	--
COOH*	-593.686	0.537	0.271	-593.420	0.240	-1.998
CO*	-582.998	0.119	0.158	-583.037	-0.228	-0.979
H*	-570.676	0.097	0.040	-570.619	0.042	-2.344
Mn-N_4	-428.901	--	--	-428.901	--	--
COOH*	-454.739	0.549	0.237	-454.439	0.905	-1.367
CO*	-444.190	0.119	0.164	-444.235	-0.406	-0.487
H*	-432.073	0.119	0.011	-431.965	0.380	-2.057

Supplementary Notes

Supplementary Note 1 | To confirm the exact structure of Mn-C_3N_4, DFT calculations were performed. Due to the incomplete condensation of g-C_3N_4 during synthesis, an imperfect composition ($C_3N_4H_{1.5}$) is usually obtained, and this structure always represent the g-C_3N_4.[1, 26, 27] The optimized lattice parameters of pure g-C_3N_4 are a = 12.7 Å, b = 16.9 Å which is slightly larger than the experimental values (a = 12.4 Å, b = 16.7 Å).[28] The DFT result implies that the Mn-N_4 configuration with four-fold Mn-N coordination is the most stable structure and the average bond length of Mn-N is 2.03Å, as shown in Supplementary Table 3 I. However, the quantitative EXAFS curve fitting analysis result confirms that the Mn-N coordination number of Mn-C_3N_4/CNT is about 3.2 (Mn-N_3) and the average bond length of Mn-N is 2.21Å, which contradicts the result of DFT calculation. The XRD results indicate that doped Mn atoms enlarge the distance among the melon units (Supplementary Figure 16). Therefore, the lattice parameter was gradually expanded from a = 12.7 Å and b = 16.9 Å to a = 13.0 Å and b = 17.6 Å and the resulted total energy and the structure of Mn-C_3N_4 at different lattice parameters are shown in Supplementary Table 3. In the optimized Mn-C_3N_4 structure, the lattice parameter is a = 12.8 Å and b = 17.6 Å, and the Mn-N coordination number is 3 with average bonding distance of 2.11 Å (Supplementary Table 3 VIII), which is basically consistent with the results of EXAFS.

Supplementary References

1. Kang, Y. et al. Selective breaking of hydrogen bonds of layered carbon nitride for visible light photocatalysis. *Adv. Mater.* **28**, 6471-6477 (2016).
2. Zhang, P., Li, X., Shao, C. & Liu, Y. Hydrothermal synthesis of carbon-rich graphitic carbon nitride nanosheets for photoredox catalysis. *J. Mater. Chem. A* **3**, 3281-3284 (2015).
3. Ju, W. et al. Understanding activity and selectivity of metal-nitrogen-doped carbon catalysts for electrochemical reduction of CO_2. *Nat. Commun.* **8**, 944 (2017).
4. Varela, A. S., Sahraie, N. R., Steinberg, J., Ju, W., Oh, H-S. & Strasser, P. Metal-doped nitrogenated carbon as an efficient catalyst for direct CO_2 electroreduction to CO and hydrocarbons. *Angew. Chem. Int. Ed.* **54**, 10758-10762 (2015).
5. Pan, F. et al. Identification of champion transition metals centers in metal and nitrogen-codoped carbon catalysts for CO_2 reduction. *Appl. Catal. B-Environ.* **226**, 463-472 (2018).
6. Zhang, B. et al. Manganese acting as a high-performance heterogeneous electrocatalyst in carbon dioxide reduction. *Nat. Commun.* **10**, 2980 (2019).
7. Zhu, Q. et al. Electrochemical reduction of CO_2 to CO using graphene oxide/carbon nanotube electrode in ionic liquid/acetonitrile system. *Sci. China Chem.* **59**, 551-556 (2016).
8. Chen, T-Y. et al. Selection of low-cost ionic liquid electrocatalyst for CO_2 reduction in propylene carbonate/tetrabutylammonium perchlorate. *ChemElectroChem* **5**, 2295-2300 (2018).
9. Zhu, W. et al. Active and selective conversion of CO_2 to CO on ultrathin Au nanowires. *J. Am. Chem. Soc.* **136**, 16132-16135 (2014).
10. Lu, Q. et al. A selective and efficient electrocatalyst for carbon dioxide reduction. *Nat. Commun.* **5**, 3242 (2014).
11. Gao, D. et al. Size-dependent electrocatalytic reduction of CO_2 over Pd nanoparticles. *J. Am. Chem. Soc.* **137**, 4288-4291 (2015).
12. Yang, F. et al. Highly efficient CO_2 Electroreduction on ZnN_4 -based single-atom catalyst. *Angew. Chem. Int. Ed.* **57**, 12303-12307 (2018).
13. Chen, Z., Mou, K, Yao, S. & Liu, L. Zinc-coordinated nitrogen-codoped graphene as an efficient catalyst for selective electrochemical reduction of CO_2 to CO. *ChemSusChem* **11**, 2944-2952 (2018).
14. Zhang, X. et al. Highly selective and active CO_2 reduction electrocatalysts based on cobalt phthalocyanine/carbon nanotube hybrid structures. *Nat. Commun.* **8**, 14675 (2017).
15. Zhu, M., Chen, J., Huang, L., Ye, L., Xu, J. & Han, Y, F. Covalently grafting cobalt porphyrin onto carbon nanotubes for efficient CO_2 electroreduction. *Angew. Chem. Int. Ed.* **58**, 6595-6599 (2019).
16. Pan, Y. et al. Design of single-atom Co-N_5 catalytic site: a robust electrocatalyst for CO_2 reduction with nearly 100% CO selectivity and remarkable stability. *J. Am. Chem. Soc.* **140**, 4218-4221 (2018).

17. Song, L. et al. Covalent organic frameworks comprising cobalt porphyrins for catalytic CO_2 reduction in water. *Science* **349**, 1208-1213 (2015).
18. Kramer, W. W. & McCrory, C. C. L. Polymer coordination promotes selective CO_2 reduction by cobalt phthalocyanine. *Chem. Sci.* **7**, 2506-2515 (2016).
19. Zhao, C. et al. Ionic exchange of metal-organic frameworks to access single nickel sites for efficient electroreduction of CO_2. *J. Am. Chem. Soc.* **139**, 8078-8081 (2017).
20. Yang, H. et al. Atomically dispersed Ni(i) as the active site for electrochemical CO_2 reduction. *Nat. Energy* **3**, 140-147 (2018).
21. Yang, J. et al. In situ thermal atomization to convert supported nickel nanoparticles into surface-bound nickel single-atom catalysts. *Angew. Chem. Int. Ed.* **57**, 14095-14100 (2018).
22. Pan, F. et al. Unveiling active sites of CO_2 reduction on nitrogen-coordinated and atomically dispersed iron and cobalt catalysts. *ACS Catal.* **8**, 3116-3122 (2018).
23. Choi, J. et al. Energy efficient electrochemical reduction of CO_2 to CO using a three-dimensional porphyrin/graphene hydrogel. *Energ. Environ. Sci.* **12**, 747-755 (2019).
24. Zhang, H. et al. Graphene supported single-atom FeN_5 catalytic site for efficient electrochemical CO_2 reduction. *Angew. Chem. Int. Ed.* **58**, 14871-14876 (2019).
25. Zhang, E. et al. Bismuth single atoms resulting from transformation of metal-organic frameworks and their use as electrocatalysts for CO_2 reduction. *J. Am. Chem. Soc.* **141**, 16569-16573 (2019).
26. Niu, P., Yin, L-C., Yang, Y-Q., Liu, G. & Cheng, H-M. Increasing the visible light absorption of graphitic carbon nitride (melon) photocatalysts by homogeneous self-modification with nitrogen vacancies. *Adv. Mater.* **26**, 8046-8052 (2014).
27. Niu, P., Liu G. & Cheng, H-M Nitrogen vacancy-promoted photocatalytic activity of graphitic carbon nitride. *J. Phys. Chem. C* **116**, 11013-11018 (2012).
28. Lotsch, B. V. et al. Unmasking melon by a complementary approach employing electron diffraction, solid-state NMR spectroscopy, and theoretical calculations-structural characterization of a carbon nitride polymer. *Chemistry* **13**, 4969-4980 (2007).
